# Data on security implications of the adoption of Internet of Things by public relations professionals

**DOI:** 10.1016/j.dib.2019.104663

**Published:** 2019-10-15

**Authors:** Lanre Amodu, Oscar Odiboh, Suleimanu Usaini, Darlynton Yartey, Thelma Ekanem

**Affiliations:** Department of Mass Communication, Covenant University, Nigeria

**Keywords:** Public relations, Internet of things, Security, Business

## Abstract

The dataset is on public relations professionals' views on the security issues related to the adoption of the Internet of Things (IoT) for the activities. The data were generated through the administration of online questionnaire to 100 public relations professionals in Nigeria and were analyzed using the Analysis of Variance (ANOVA).

**Table:** Specifications Table

Subject area	*Public Relations*
More specific subject area	*Public relations and Information Communications Technologies*
Type of data	*Primary data (Tables)*
How data was acquired	*Survey through the use of a questionnaire*
Data format	*Raw and analyzed with Statistical Package for Social Sciences*
Experimental factors	*The data for the survey was obtained from one hundred respondents that were practicing public relations in Nigeria. The instrument- questionnaire- was designed by the researchers and ANOVA was used to analyze the data.*
Experimental features	*Public relations is a profession that manages relationships and information. While the adoption of IoT is valuable, it is necessary to examine cyber security issues.*
Data source location	*Nigeria*
Data accessibility	*Dataset is with this article*

**Table:** 

**Value of the Data** 1. The data can serve as a basis for further research of the use of the Internet of Things for public relations activities. 2. The analyzed data can reveal factors that can influence public relations professionals' perception of security issues with regard to the adoption of IoT. 3. The outcome of the data can assist in showing what can affect the public relations professionals' interest in the adoption of IoT.

## 1 Data

Public relations is generally defined as a management function that establishes and maintains mutually beneficial relationship between an organisation and its relevant publics [1,2]. To build relationships, exchange of information is essential [3]. Several scholars have explored public relations' use of the Internet and social media platforms for better stakeholders engage [[4], [5], [6], [7]]. There is, however, dearth of research on the potentials of the adoption of the Internet of Things (IoT) for public relations. IoT had been described as the interconnectivity of things online in such a way that they interact without human interference [8]. It involves giving “senses” to objects that are online to enable them generate and transmit data. While the practice of public relations online still elicits fear of the security implications of managing sensitive information on the cyber space, the fear is likely to be more significant in the face of having several datapoints interconnected, which suggests that an unauthorized access to one is potentially an access to all.

The dataset is on factors that can influence public relations professionals' perception of security issues relating to the adoption of IoT. The survey research adopted snowball technique to sample the opinion of respondents. Analysis of Variance (ANOVA) was used to test the factors that may ultimately affect the professionals' interest in IoT. Table 1, Table 2, Table 3, Table 4, Table 5, Table 6 present the results of the analysis. Considering that the use of IoT in public relations is largely an unexplored area, this data is relevant for academic use by extending the frontiers of research and prompting crucial conversations on it. The data are also useful to managements of public relations organisations and businesses with in-house public relations units as they make critical decisions with regard to technology adoption.

**Table 1:** Gender distribution.

Gender	Percentage (%)
Male	52
Female	48
Total	100 n = 100

**Table 2:** Age distribution.

Age	Percentage (%)
20–30	53
31–40	18
41 and above	29
Total	100 n = 100

**Table 3:** Organisational distribution.

Organisation Type	Percentage (%)
In-house Public Relations Unit	64
Independent Public Relations Firm	36
Total	100 n = 100

**Table 4:** ANOVA.

Model		Sum of Squares	df	Mean Square	F	Sig.
1	Regression	.662	5	.132	.556	.733
	Residual	22.378	94	.238		
	Total	23.040	99

**Table 5:** ANOVA.

Model		Sum of Squares	df	Mean Square	F	Sig.
1	Regression	18.360	5	3.672	3.459	.007
	Residual	99.800	94	1.062		
	Total	118.160	99

**Table 6:** ANOVA.

Model		Sum of Squares	df	Mean Square	F	Sig.
1	Regression	43.491	5	8.698	3.223	.010
	Residual	253.719	94	2.699		
	Total	297.211	99			

## 2 Experimental design, materials and methods

The data were generated from an online survey conducted among 100 public relations professionals. The snowball sampling technique was used to select the respondents. The research was carried out over six months in which major online platforms of public relations associations and organisations were used to invite participation. It was, however, observed that several professionals and even association members preferred to fill hard copies of the questionnaire. This request could not be accommodated in the research considering that the subject is ICT related and online participation is considered a precondition for involvement in the study. This factor, therefore, affected the number of respondents for the research. The questionnaire used for the data collection was designed by the researchers specifically to elicit response on the variables under consideration. Statistical Package for Social Science (SPSS) was used to analyze the data.

## Ethical considerations

The researchers ensured that participants in the research were aware of the purpose of the data gathering and how the data will be used. Their anonymity was guaranteed by using a Google form that was devoid of any personal identification of the respondents. They were also assured that the data were strictly for research purposes and their responses would be kept confidential.
