# Supplementary material for: Data on security implications of the adoption of Internet of Things by public relations professionals
Source: Data Brief. 2019 Oct 15;27:104663. doi: 10.1016/j.dib.2019.104663 (PMC6838381; doi:10.1016/j.dib.2019.104663)
Supplement: Multimedia component 2 [file mmc2.docx]

QUESTIONNAIRE

Dear Respondent,

This is a survey on ***The Implications of Internet of Things for Public Relations***. You are kindly requested to fill the questionnaire based on your candid option on the subject. This instrument is solely for research and your response will be treated with complete confidentiality. It would be deeply appreciated if you don’t leave any question unanswered. Thank you for your kind attention.

Best regards

Section A: Demographic

1. Gender a) Male c) Female
2. Age a) 20-30 b) 31-40 c) 41-50 d) 51 and above
3. Organisational type a) Independent PR Firm b) In-House PR Unit/Department within an organisation c) Other
4. Organisation’s state of operation ------------------------------------
5. Rough estimate of your organisation’s customer/client size a) 100 and below b) 101-400 c) 401-700 d) 701-1000 e) 1001 and above
6. Your years of service in your current organisation a) 0-5 b) 6-10 c) 11-15 d) 16 and above
7. Your years of experience in the Public Relations industry a) 0-5 b) 6-10 c) 11-15 d) 16-20 e) 21 and above

Section B: Awareness

| S/N | Items | SA | A | U | D | SD |
| --- | --- | --- | --- | --- | --- | --- |
| 8 | I have heard of the “Internet of Things” (IOT) |  |  |  |  |  |
| 9 | IOT is the interconnectivity of objects to objects |  |  |  |  |  |
| 10 | The Internet is essential for IOT to be functional |  |  |  |  |  |
| 11 | IOT involves giving senses to objects to collect and transmit data |  |  |  |  |  |
| 12 | IOT is a software |  |  |  |  |  |

Section C: Interest/Potentials/Possibilities

| S/N | Items | SA | A | U | D | SD |
| --- | --- | --- | --- | --- | --- | --- |
| 13 | I will like to control equipment in my office (Computers, temperature, printer, surveillance, etc) from my mobile device. |  |  |  |  |  |
| 14 | I will like to access all data relating to my business on my mobile device. |  |  |  |  |  |
| 15 | I will like to receive real-time data from my customer/client on how to improve my services |  |  |  |  |  |
| 16 | I will like to receive real-time data on my customer/clients’ complaints. |  |  |  |  |  |
| 17 | I will like to increase channels of reaching my customer/client (eg. Phone, smart watch, digital vehicular displays, etc). |  |  |  |  |  |
| 18 | My public relations decisions are made based on data. |  |  |  |  |  |
| 19 | I will like to engage my customer/client directly without media interface |  |  |  |  |  |
| 20 | I will appreciate a technology that can help me avoid crisis by analyzing the trend of my customer/clients’ complaints |  |  |  |  |  |
| 21 | I will appreciate a technology that can predict my customers/clients’ needs. |  |  |  |  |  |
| 22 | I will appreciate a technology that can help me customize my messages to each customer/client. |  |  |  |  |  |

Section D: Security

| S/N | Items | SA | A | U | D | SD |
| --- | --- | --- | --- | --- | --- | --- |
| 23 | I have no reservation about connecting all my devices to the internet |  |  |  |  |  |
| 24 | I am not afraid of information leak on the internet |  |  |  |  |  |
| 25 | I am not afraid of vital organisational database being hacked into through the Internet. |  |  |  |  |  |
| 26 | My organisation has software protection against hacking |  |  |  |  |  |
| 27 | I believe it is ethical to access as much data as possible about my customer/client through any medium |  |  |  |  |  |
| 28 | The more I know about my customers/clients, the better I can satisfy them. |  |  |  |  |  |
| 29 | I believe I have the responsibility to protect my customers/clients’ data. |  |  |  |  |  |
| 30 | I will appreciate a technology that can give me access to my customers/clients’ data without their permission. |  |  |  |  |  |

Section E: Readiness

| S/N | Items | SA | A | U | D | SD |
| --- | --- | --- | --- | --- | --- | --- |
| 31 | I believe power supply will be a limitation to the interconnectivity of devices online. |  |  |  |  |  |
| 32 | I believe Internet connection will be a limitation to the interconnectivity of devices online. |  |  |  |  |  |
| 33 | I believe security will be a limitation to the interconnectivity of devices online. |  |  |  |  |  |
| 34 | I believe general acceptance will be a limitation to the interconnectivity of devices online. |  |  |  |  |  |
| 35 | I see my organisation interconnecting all its devices through the Internet in the next 5 years. |  |  |  |  |  |
| 36 | I see my organisation adopting technology that can gather customers/clients’ real-time data in the next 5 years. |  |  |  |  |  |

https://goo.gl/forms/CmgaW7lhVc0HsSBB2
